# Supplementary material for: Engineering Genetically-Encoded Mineralization and Magnetism via Directed Evolution
Source: Sci Rep. 2016 Nov 29;6:38019. doi: 10.1038/srep38019 (PMC5126674; doi:10.1038/srep38019)
Supplement: Supplementary Information [file srep38019-s1.pdf]

Engineering Genetically-Encoded Mineralization and Magnetism via Directed Evolution

Xueliang Liu[1,2,3], Paola A. Lopez[4], Tobias W. Giessen[1,3], Michael Giles[5], Jeffrey C. Way[1,3], Pamela A. Silver*[1,3]

[1] Wyss Institute for Biologically Inspired Engineering;
[2] School of Engineering and Applied Sciences, Harvard University;
[3] Department of Systems Biology, Harvard Medical School;
[4] Graduate Program in Bioengineering UC Berkeley/UCSF;
[5] Harvard College, Harvard University

To whom correspondence should be addressed:

Pamela A. Silver, PhD
Elliot T and Onie H Adams Professor of Biochemistry and Systems Biology
Harvard Medical School
Department of Systems Biology
200 Longwood Avenue
Warren Alpert Building
Boston, MA 02115
Phone: 617-432-6401
Fax: 617-432-5012
Email: pamela_silver@hms.harvard.edu

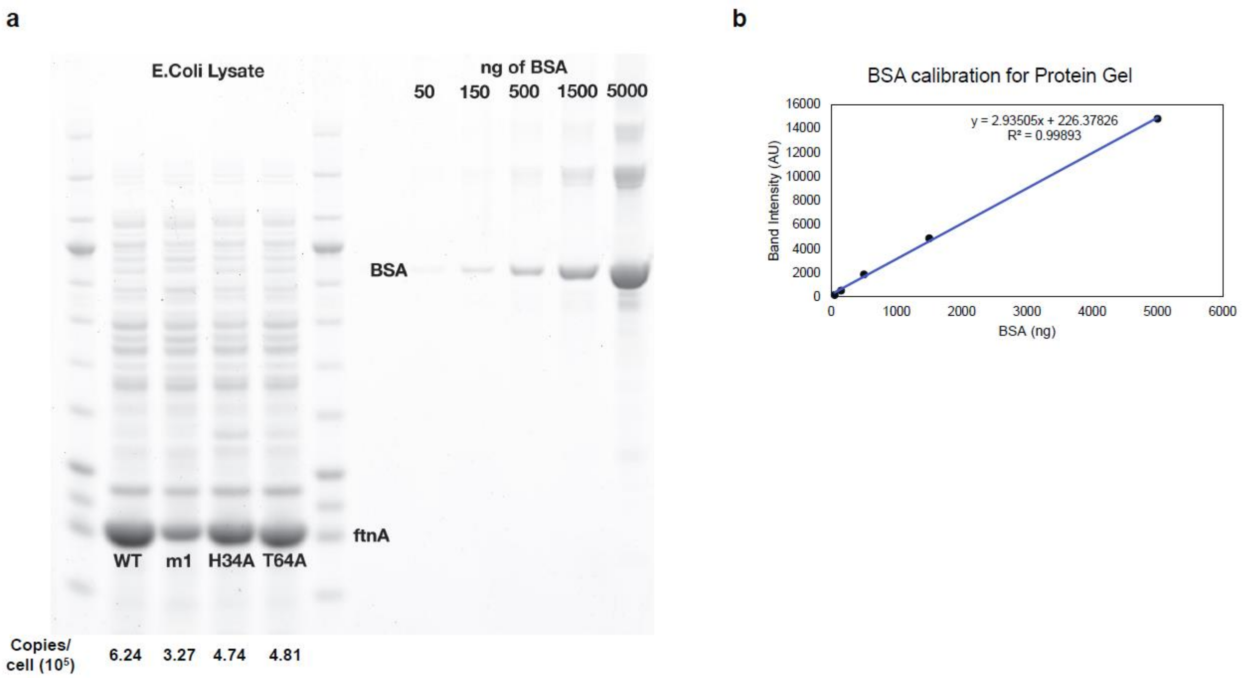

Figure S1 BSA-calibrated SDS-PAGE gel shows lower ferritin expression level for mutants. (a) SDS-PAGE gel of cell lysate from ferritin over-expressing E. coli shows strongest expression for wildtype (WT) compared to the more magnetic ferritin mutants. **(b)** Band intensities versus amount of BSA loaded (in ng) produces good linear fit (R^2 = 0.999). The fit parameters were used along with culture OD and assumed 5×10^8 cells per ml concentration at OD_{600} = 1 to calculate the protein copies per cell in A.

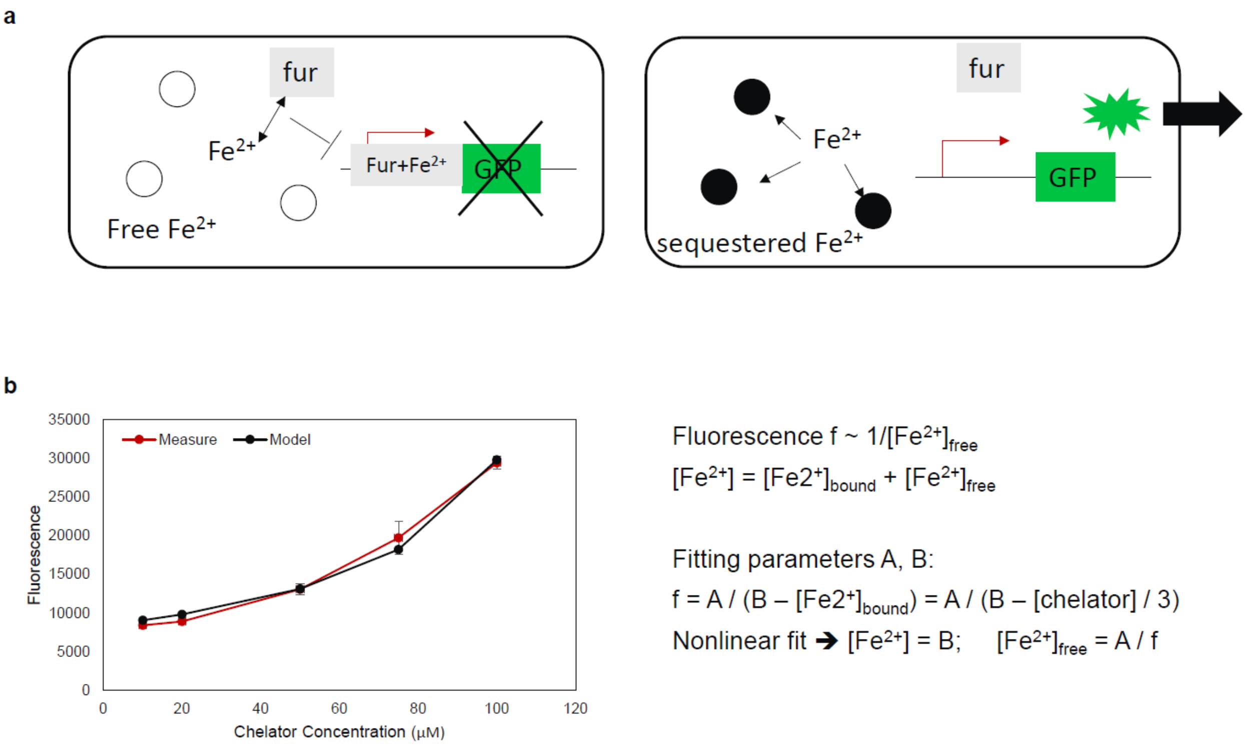

Figure S2 Novel fluorescent genetic sensor for cytoplasmic free Fe^{2+}. (a) Free Fe^{2+} binds to the ferric uptake regulator (apo-fur). The Fe-bound fur binds the fiu promoter sequence to repress transcription of GFP (right). Sequestration of free Fe^{2+} by ferritins increases sensor fluorescence output. **(b)** The concentration of free Fe^{2+} can be deduced from the fluorescence signal by calibration of sensor output with titration of known concentrations of Fe^{2+} chelator bipyridine. The simple nonlinear model produces good fit to the measured fluorescence.

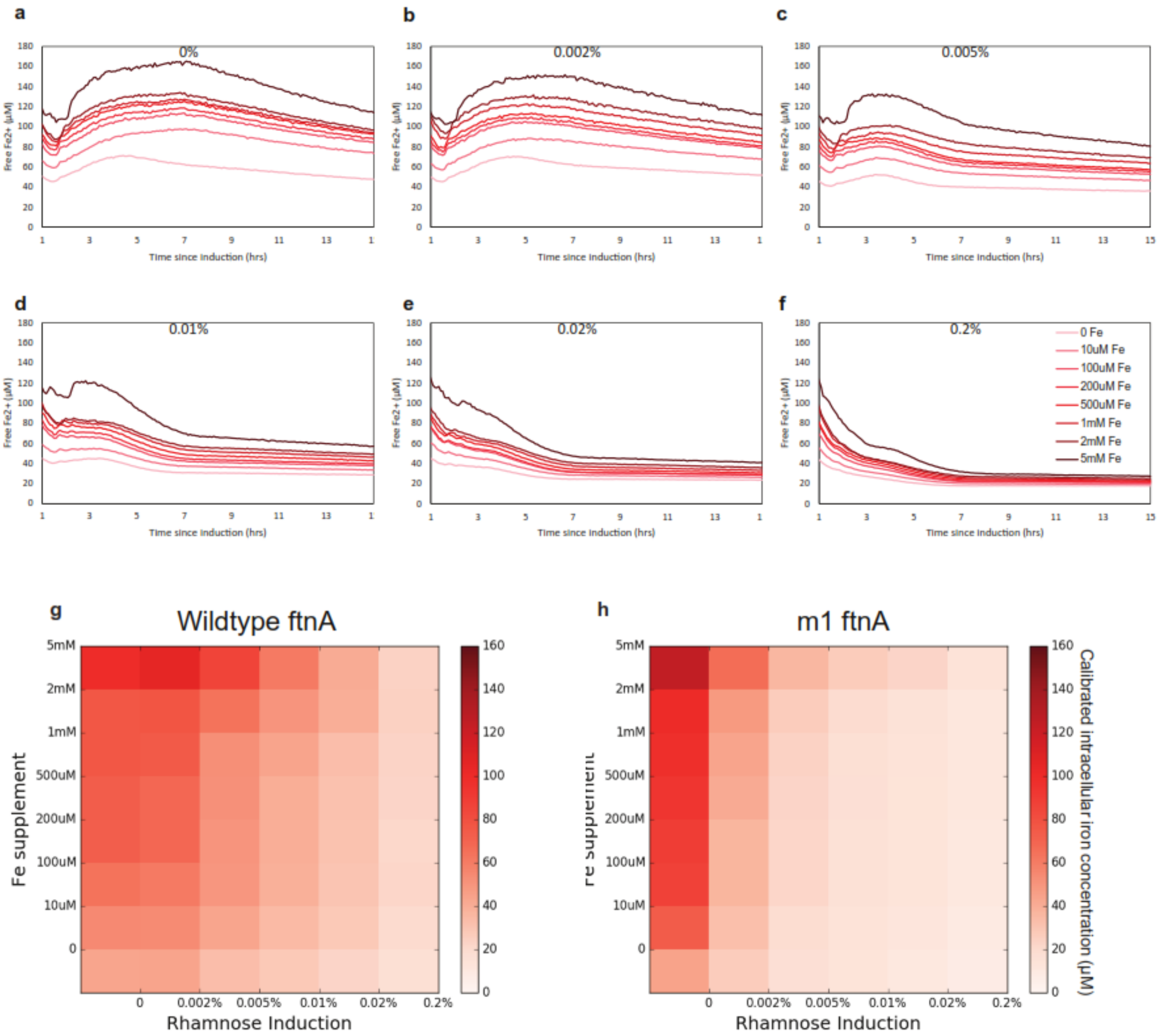

Figure S3 Genetic fluorescent sensor monitors cellular Fe^{2+} sequestration in WT and mutant ferritin expressing cells (second replicate of data in Figure 3). **(a-f)** Calibrated free Fe^{2+} concentrations in *E. coli* expressing wildtype ferritin from up to 15 hours after induction by 0% (a), 0.002% (b), 0.005% (c), 0.01% (d), 0.02% (e), and 0.2% (f) rhamnose with media Fe^{2+} supplement of 0 µM, 10 µM, 100 µM, 200 µM, 500 µM, 1 mM, 2 mM, 5 mM. Without ferritins high media supplement at 5mM can dramatically alter the intracellular iron homeostatic setpoint. At high induction levels free Fe^{2+} is efficiently sequestered up to the highest media supplement concentration. The heatmaps with color saturation proportional to calibrated intracellular free

iron levels show that compared to the wildtype **(g)**, the best ferritin mutant **(h)** is much more effective at sequestering iron at lower protein levels (0.01% rhamnose induction) and at high environmental iron concentration (up to 2 mM). This is consistent with their greater magnetism despite lower protein expression.

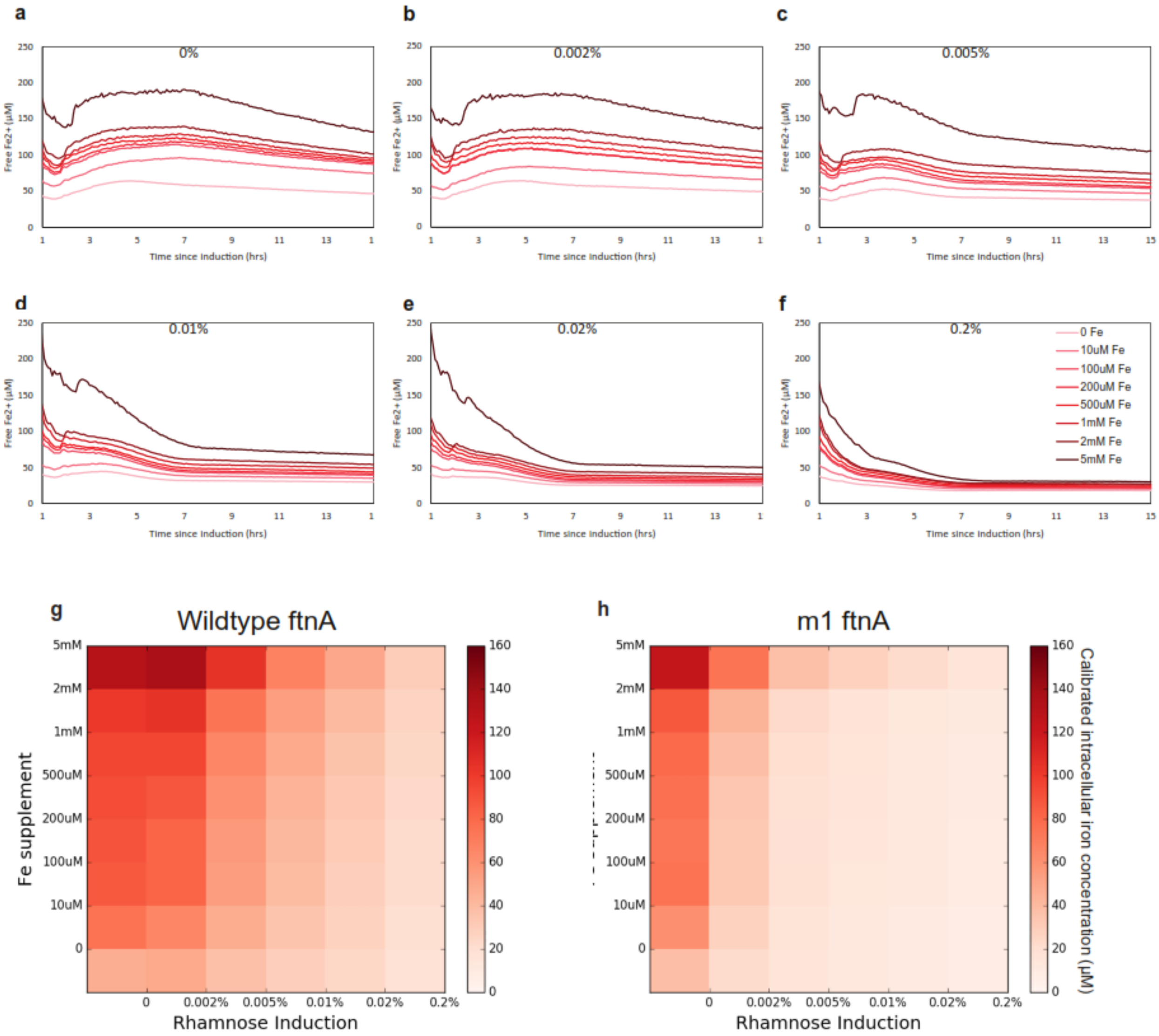

Figure S4 Genetic fluorescent sensor monitors cellular Fe^{2+} sequestration in WT and mutant ferritin expressing cells (third replicate of data in Figure 3). (a-f) Calibrated free Fe^{2+} concentrations in *E. coli* expressing wildtype ferritin from up to 15 hours after induction by 0% (a), 0.002% (b), 0.005% (c), 0.01% (d), 0.02% (e), and 0.2% (f) rhamnose with media Fe^{2+}

supplement of 0 µM, 10 µM, 100 µM, 200 µM, 500 µM, 1 mM, 2 mM, 5 mM. Without ferritins high media supplement at 5mM can dramatically alter the intracellular iron homeostatic setpoint. At high induction levels free Fe^{2+} is efficiently sequestered up to the highest media supplement concentration. The heatmaps with color saturation proportional to calibrated intracellular free iron levels show that compared to the wildtype **(g)**, the best ferritin mutant **(h)** is much more effective at sequestering iron at lower protein levels (0.01% rhamnose induction) and at high environmental iron concentration (up to 2 mM). This is consistent with their greater magnetism despite lower protein expression.

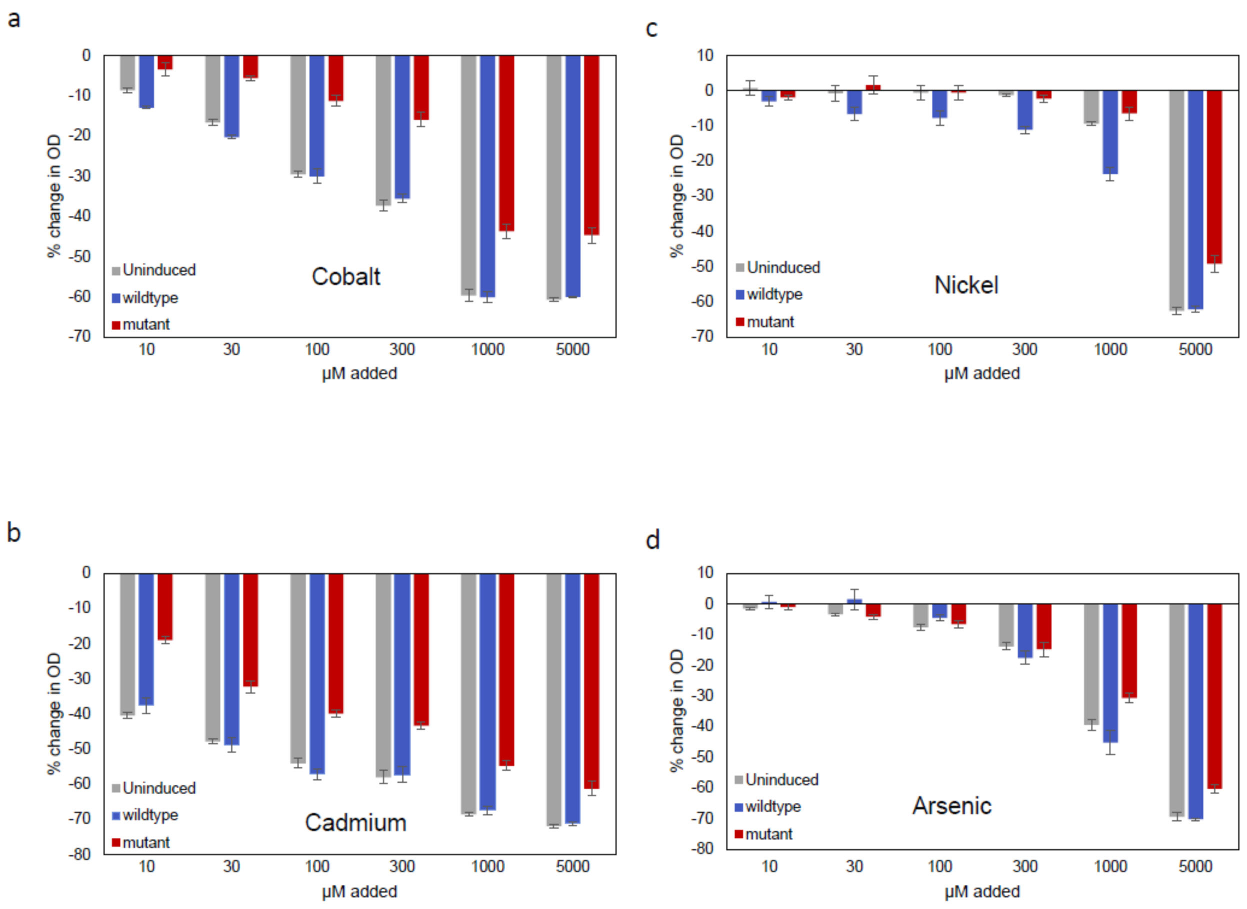

Figure S5 Magnetic mutant ferritin over-expression reduces cellular growth defect. Percent change in OD was measured in early saturation phase for cells expressing no (grey), wildtype (blue) or magnetic mutant (red) ferritins and incubated in LB media containing between 0 and 5mM of cobalt (a), cadmium (b), nickel (c) and arsenic (d). Expressing the magnetic mutant ferritin exhibited the least growth defect whereas expressing no or the wildtype ferritins had similarly strong growth defect. The effect is most pronounced at low concentrations for cadmium and cobalt.

m1	H34L+T64I
m2	SpyTag(N-term)+R56P
m3	F58L+T116R
m4	SpyTag(N-term)+T64I
m5	SpyTag(N-term)+A47T
m6	T64I+T116R
m7	R56P+H128R
m8	SpyTag(N-term)+F58L
m9	SpyTag(N-term)+H34A+T64A+F58A
m10	SpyTag(N-term)+H128R
m11	T116R
m12	F58L+H128R
m13	T64I
m14	T116R+H128R
m15	T64I+H128R
m16	H128R
m17	F58A
m18	SpyTag(N-term)
m19	T64I+F58L
m20	SpyTag(N-term)+T116R
m21	F58L
m22	A47T
m23	H34A+T64A+F58A
m24	H128A
m25	R56P
m26	H34A
m27	T64A
m28	L18Q
m29	K140A
m30	L104Q
m31	R56A
m32	K156A
m33	H34A+T64A+F58A+T116R+H128R
m34	H34A+T64A+F58A+T116R
m35	R56P+T116R
m36	L10P
m37	SpyTag(N-term)+H34A+T64A+F58A+T116R+H128R
m38	SpyTag(N-term)+H34A+T64A+F58A+T116R

Table S1 List of ferritin mutants characterized with iron sensor and magnetic column
